# Quantifying Memory in Complex Physiological Time-Series

**DOI:** 10.1371/journal.pone.0072854

**Published:** 2013-09-05

**Authors:** Amir H. Shirazi, Mohammad R. Raoufy, Haleh Ebadi, Michele De Rui, Sami Schiff, Roham Mazloom, Sohrab Hajizadeh, Shahriar Gharibzadeh, Ahmad R. Dehpour, Piero Amodio, G. Reza Jafari, Sara Montagnese, Ali R. Mani

**Affiliations:** 1 Computational Physical Sciences Research Laboratory, School of Nano-Science, Institute for Research in Fundamental Sciences (IPM), Tehran, Iran; 2 Department of Physiology, Faculty of Medical Sciences, Tarbiat Modares University, Tehran, Iran; 3 Department of Medicine, University of Padova, Padova, Italy; 4 Neuromuscular Systems Laboratory, Faculty of Biomedical Engineering, Amirkabir University of Technology, Tehran, Iran; 5 Department of Pharmacology, School of Medicine, Tehran University of Medical Sciences, Tehran, Iran; 6 Department of Physics, Shahid Beheshti University, Tehran, Iran; Université de Montréal, Canada

## Abstract

In a time-series, memory is a statistical feature that lasts for a period of time and distinguishes the time-series from a random, or memory-less, process. In the present study, the concept of “memory length” was used to define the time period, or scale over which rare events within a physiological time-series do not appear randomly. The method is based on inverse statistical analysis and provides empiric evidence that rare fluctuations in cardio-respiratory time-series are ‘forgotten’ quickly in healthy subjects while the memory for such events is significantly prolonged in pathological conditions such as asthma (respiratory time-series) and liver cirrhosis (heart-beat time-series). The memory length was significantly higher in patients with uncontrolled asthma compared to healthy volunteers. Likewise, it was significantly higher in patients with decompensated cirrhosis compared to those with compensated cirrhosis and healthy volunteers. We also observed that the cardio-respiratory system has simple low order dynamics and short memory around its average, and high order dynamics around rare fluctuations.

## Introduction

The study of physiological rhythms (e.g. respiration, cardiac cycles) and their regulation using reductionistic methods has provided a comprehensive body of knowledge on physiological systems after different types of interventions. However, the limitation of this approach is that the original system needs to be disrupted. Thus, instead of describing the original system, we study a perturbed system that may or may not display the features of the original system. Thus, there is a need to characterize the complexity of physiological regulation without intervention on or isolation of its different components [1], [2].

Physiological mechanisms underlying cardio-respiratory variations include “deterministic” multiple feedback loops regulating the cardio-respiratory system, as well as “stochastic” processes at the cellular and molecular levels (e.g. ion channels, neurotransmitter release etc) [3]. The stochastic nature of real systems precludes the use of deterministic models to describe physiological variations. Thus, stochastic methods may provide useful information on the complexity of physiological rhythms, and uncover mechanisms which are associated with complex pathologies such as cardiac arrhythmia and asthma.

One way to approach complexity by stochastic methods is looking for the presence of Markov property, which can be detected in natural systems above a certain time or length scale [4], [5]. Intuitively, the physical interpretation of a Markov process is that it is a process that ‘forgets its past’. In other words, the ability to predict its value at any given time is not enhanced by knowing its values in steps prior the most recent one [4]. In real complex systems (e.g. biological rhythms) it is difficult to find absolute Markov processes but Markov properties may be expected to hold for a time scale (Markov length) that is the time scale over which the process can be thought of as a Markov process [4]. The Markov length of a time-series shows how many steps in the time-series we need to go forward to reach a point at which the present state of the system does not depend on its past [4]–[8]. In this context, the calculation of such time scale gives us information on the memory of a complex time-series about its past. Recent studies have shown that these calculations provide useful results for such diverse fields as turbulence, seismic wave analysis and finance [4], [6]–[8]. Their use in physiological time-series may also provide novel insights (e.g. memory) that have not been described using classical, reductionistic methods.

Although short-term memory has been addressed in cognitive neuroscience, this concept has not adequately been explored within the context of autonomic physiological rhythms, such as cardio-respiratory fluctuations. Ghasemi *et al*. calculated Markov time scale in cardiac inter-beat interval fluctuations from 8-hour electrocardiogram (ECG) in healthy subjects and patients with congestive heart failure, and showed that Markov length is significantly lower in healthy people compared to patients with heart failure [9]. This indicates that each heartbeat keeps a longer memory of previous heartbeats in patients with heart failure compared to healthy individuals, a phenomenon which may be a disadvantage in terms of responding to an ever-changing, complex environment. Although several methods have been proposed for detecting Markov properties in time-series (e.g. Chapman-Kolmogrov method) [4], they usually require long time-series, which are not always available for purposes of physiological and clinical investigations.

We have recently applied a method called “inverse statistics” to study heart rate dynamics in both health and disease [10]. Inverse statistics has extensively been used to study turbulence and financial markets, and uncovers the most likely waiting time needed to reach a certain change in a time-series [11]–[15]. In the heartbeat time-series, it is used to study the distribution of waiting times needed to reach a rare event in heart rate [10]. This analysis also showed that inverse statistical analysis can distinguish between the ECG taken from healthy volunteers and patients with congestive heart failure. Moreover, the inverse statistical analysis suggested that it is more likely that a rare event (e.g. bradycardia or tachycardia) is followed by further rare events in pathological situations, such as congestive heart failure [10]. This type of dynamics has qualitatively been reported in many pathologic conditions, such as ventricular tachycardia and air trapping during asthma attacks [16]–[18]. However, there is a need to develop a method to evaluate such dynamics quantitatively. In this paper, we describe inverse statistics as a method for analyzing non-stationary physiological data that provides information on memory in cardio-respiratory time-series. We also show the application of this method to the analysis of respiratory and heart rate fluctuations in patients with asthma and liver cirrhosis, respectively.

## Methods

### A. Theory

**Figure 1:**
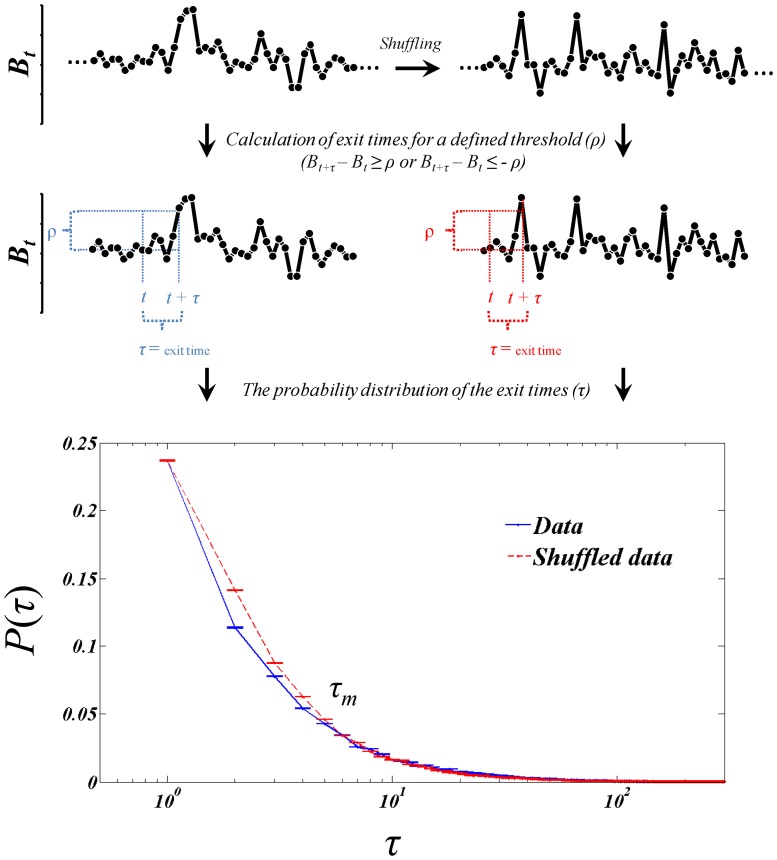
Schematic view of the algorithm used to obtain the probability distribution of the exit times (τ) needed to observe accelerating or decelerating events for a defined threshold (ρ) in the original as well as the shuffled time-series (B_t_).

Time-series are a collection of observations gathered over time. For example, suppose {*B_1_*, *B_2_, B_3_*, … } are inter-beat intervals of consecutive cardiac cycles recorded from an individual. Such set of data can be plotted against time as shown in figure 1. Physiological time-series often exhibit a complex dynamics and the interest of time-series analysis is to see what these consecutive data can tell us about the underlying dynamics of the system. Inverse statistical approach deals with the probability to observe a jump in a time-series [10]–[14]. A jump at a predefined level (ρ) means finding the points in a time-series that the inter-beat interval becomes ρ second slower (or faster) than expected. In order to describe this approach we firstly need to define “exit time” in a time-series (definitions of the technical terms are given in appendix I). Exit time is defined as the waiting time (*τ*) needed to achieve a predefined difference in a time-series. Inverse statistics is a technique which is based on exit time distribution of a stochastic process [10]–[14]. The method was originally developed for the analysis of turbulence [11] and after that, it has been used in time-series analysis in various disciplines [10], [12]–[15].

Given a time-series *B_t_*, the exit time *τ* is the minimum time needed to see the jump *B_t+Δt_−B_t_ = ΔB = ρ* (figure 1). Using this criteria, we can construct a new time-series *τ* and all statistical variables measured in the new time-series give “inverse” information compared to classical statistical parameters. A well-known measure in this context is the distribution of the new time-series, which means the distribution of exit times in the original time-series. Although it seems that the original time-series and the inverted one are related to each other, it has already been shown that they are independent [11]. This guarantees that inverse statistics provide novel insight into the physiological time-series compared to conventional analytical methods.

One of the most prominent results of this technique is comparing the exit time distribution of the main process and its shuffled version [10]. As the shuffling process disrupts the order of data, it tends to keep the probability distribution function but it destroys any time correlation within the series. Shuffling of a time-series should be performed in return (derivative) of data which are in a stationary space [10]. After that we should make a profile (integration) of the data to return to the non-stationary form. Following this algorithm, we keep one-step joint probabilities – which define Markov process – of the time-series and delete all longer joint probabilities [10].

Now we have two time-series, the original one and the shuffled one (figure 1). We then calculate the exit time distribution in these two time-series in relation to a defined jump (*ρ*). The resulting probability distribution curves show the probability to observe an event which is *ρ* second slower (when *ΔB≥ρ*) or faster (when *ΔB≤−ρ*) than a given point in the time-series. Through this report, we define such events as “rare”. The level of ‘rarity’ clearly depends on the definition of *ρ* and it is convenient to set this level in relation to the standard deviation of the data set (σ), allowing measurements on data sets with different levels of variability to be compared.

Figure 1 shows the probability distribution curves of the exit times in both the original and shuffled time-series. Comparing these two distributions reveals deviation from a shuffled process in observing a rare event at different time scales (*τ*). It can be observed that the difference between the two distributions occurs only within small *τ* regions, and afterwards the two distributions merge. We call this interval *τ = τ_m_* ‘maximum joint probability length’ because all longer joint probabilities can be produced by the product of other, shorter joint probabilities (see appendix II).

Figure 1 shows a sample of these two distributions with an overlap after some *τ = *5. The probability of this point can be calculated from the two graphs separately. In the shuffled distribution, one can multiply the probability of transitions in each step, regardless of their temporal positions. As overlap is continued for larger *τ* times, all of the larger joint probabilities can be written as the product of shorter joint probabilities (for more description see appendix II). Thus, the shortest joint probability which is reducible to one step probability is of the order of 5. Hence, the dynamic equation of the system keeps all joint probabilities which have order <5. This means there is some coupling between data points within 5 steps. This length can be interpreted as the “memory length” of the system, which shows how many previous data affect the present data point. If we compile it to differential equation literature, it implies that the biggest derivative of the system and the order of its dynamical equation is 5.

As described above, finding the last non-overlapping point in these two distributions – main exit times and shuffled exit times – is an approximation of the order of its dynamical equation. Hence, due to control theory, the larger the interval is, the harder it will be to ‘control’ the system. Indeed, this depends on the threshold (*ρ*) which we set to define the rare event. This concept is comparable to Markov length, or the minimum scale (step) at which there is a transition from a non-Markov to a Markov process [4]. The main difference in our approach is level-dependency, which carries the information of system dynamics behavior in relation to a pre-defined level for the observation of a rare event. The Markov length calculated based on the Chapman-Kolmogrov formula, averages over all levels of fluctuation. In contrast, with our technique, the information on how the system responds within different level of fluctuation (*ρ*) in the time-series is also available. By determining this, we can study the transitions of the system in relation to the degree of its variations, and make a simple schema of the system dynamics behavior.

Another advantage of this method is that one can look at the joint probability of decelerating events (e.g. bradycardia, bradypnea) and accelerating events (e.g. tachycardia, tachypnea) separately [10]. If the exit time is defined as the time *τ* so that *B_t+τ_ –B_t_≥ρ*, the joint probability of decelerating rare events is studied. If the exit time is defined as the time *τ* so that *B_t+τ_ –B_t_≤−ρ*, one can look at the joint probability of accelerating rare events. In this paper, we provide one example by looking at accelerating rare events in inter-breath intervals in healthy volunteers and patients with different types of asthma. We also used inverse statistics approach for cardiac cycle time-series analysis in healthy people and patients with liver cirrhosis.

### B. Data collection

#### Ethics statement

All data were recorded according to the recommendations and approval of the Ethics Committees of the Tarbiat Modares University and the University of Padova. The review board or ethics committee of both mentioned universities approved the study and written informed consents were obtained from all patients and healthy volunteers.

#### Respiratory time-series study

Asthma is a major respiratory disease worldwide and is classified as atopic (extrinsic) or non-atopic (intrinsic), based on whether symptoms are precipitated by allergens (atopic) or not (non-atopic). Very little is known about the pathophysiology of non-atopic asthma, and it is usually difficult to prevent or control this type of asthma with standard remedies. Forty age-matched men including 10 healthy volunteers, 10 patients with controlled atopic asthma, 10 patients with uncontrolled atopic asthma, and 10 patients with uncontrolled non-atopic asthma, ages 21 to 39 years, referred to the outpatient clinic of Masih Daneshvari Lung Hospital (Tehran, Iran) were enrolled in this study and written informed consents were obtained. Asthma was categorized as controlled and uncontrolled based on the National Asthma Education and Prevention Program (NAEPP) guidelines [19]. Atopic asthma was diagnosed based on the results of skin tests and clinical symptoms. Subjects laid supine for about 70 min while continuous respiration signals were collected, using a respiratory inductive plethysmography. Two pneumotrace bands (AD-Instruments, Australia) were fastened at the level of umbilicus and fourth intercostal interspace, for monitoring of rib cage and abdomen movements. The signals from the pneumotrace bands were digitized at a 1 KHz sampling rate (Powerlab, AD-Instruments, Australia). The plethysmography signals were calibrated using an artificial neural network system, as previously described [20]. The inter-breath intervals were then calculated for 60 min using an *ad hoc* computer program (Chart 5, AD-Instrument, Australia).

#### Cardiac time-series study

Liver cirrhosis is associated with cardiovascular complications such as cardiomyopathy and hyperdynamic circulation, which impinge on the pathophysiology of the disease, particularly in patients with decompensated cirrhosis [21], [22]. In order to compare the inverse statistical properties and memory length of the cardiac inter-beat interval time-series in healthy subjects and patients with cirrhosis were studied. Ninety-three patients with cirrhosis referred to the outpatient clinics of the Department of Medicine of the University of Padova (Italy) and no significant co-morbidity were enrolled. The functional severity of the liver injury was assessed by using Pugh's modification of the Child's grading system [23]. Patients with Pugh scores >7 (Child B and C) were qualified as having decompensated cirrhosis. The reference population comprised 41 age- and sex-matched healthy volunteers from the Padova area. None had a history, clinical or laboratory evidence of liver or heart disease. A 10-min, single channel ECG was recorded by placing a silver-silver chloride electrode on each wrist. The ECG data were digitized at a sampling rate of 256 Hz. The R peaks were detected and the R-R interval series generated by using an *ad hoc* computer program (Chart 5, AD-Instrument, Australia). The R-R interval series was visually inspected and 8-min, artifact-free continuous R-R interval sections were selected for analysis.

#### Statistical analysis

In order to allow comparisons of data sets with different degrees of variability, all time-series were normalized to have σ = 1. We used the terminology and notation of Ebadi *et al.* [10] in describing techniques for inverse statistical analysis of physiological time-series. Inverse statistical analysis was performed using MATLAB (version 7.8.0), as described above. To compare the mean values, we used two-way ANOVA to test the difference between exit times of patients and healthy volunteers over all τ times. When two-way ANOVA gave significant outcome, Bonferroni *post-hoc* test was used to find out which group(s) differed from the rest.

## Results

### Inverse statistical approach applied to the breathing time-series in patients with asthma

The inverse statistical properties of the inter-breath interval time-series in healthy subjects and patients with asthma were investigated. In the present study, inverse statistics were applied to four groups of aged-matched subjects: 10 healthy volunteers, 10 subjects with controlled atopic asthma (CAA), 10 subjects with uncontrolled atopic asthma (UCAA) and 10 subjects with uncontrolled non-atopic asthma (UNAA).

**Figure 2:**
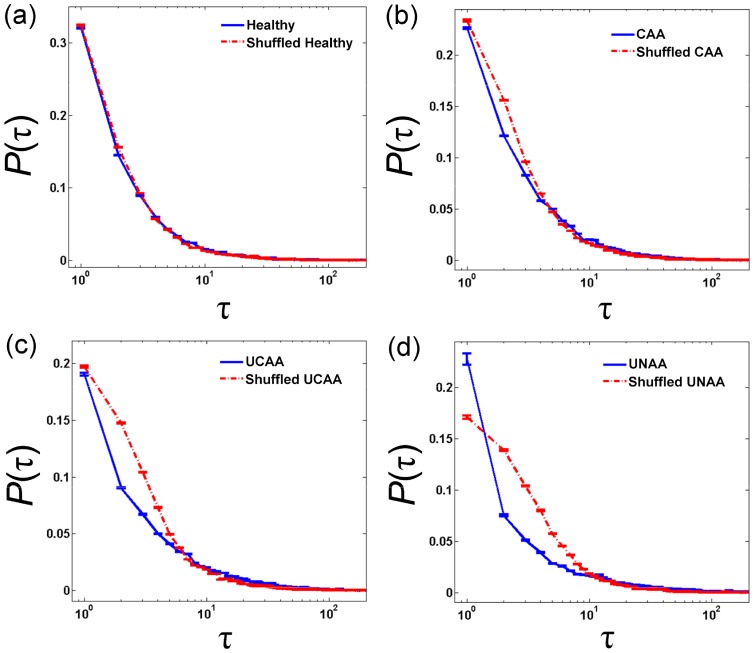
The probability distribution of the normalized exit times (*τ*) needed to observe an accelerating event that is ρ = 0.5σ second faster than a given point (t) within the time-series (ΔB = B_t+τ_−B_t_≤−0.5σ). Respiratory inter-breath interval time-series were obtained from (a) 10 healthy volunteers, (b) 10 patients with controlled atopic asthma (CAA), (c) 10 patients with uncontrolled atopic asthma (UCAA) and (d) 10 patients with uncontrolled non-atopic asthma (UNAA). Blue solid lines represent probability distribution of *τ* of the original time-series and red dash-dot lines correspond to their shuffled time-series. Data are presented as mean ± standard error of mean.

Figure 2 shows the exit times distribution for the levels of ρ = 0.5σ (σ is the standard deviation of the inter-breath interval time-series) in healthy volunteers and asthmatic patients, and the corresponding shuffled data when *ΔB≤−ρ*. Within this context, ρ = 0.5σ means finding the points that the inter-breath interval becomes ρ seconds faster than expected. Such curves indicate the probability distribution of times to observe respiratory cycles ρ seconds faster than expected. As shown in this figure, in the healthy volunteers, the probability curve soon overlaps with the shuffled data curve. In contrast, in patients with asthma there is considerably more difference between the original and shuffled sets [curves in figures 2(a) with (b), (c) and (d)]. In addition, the difference is even more profound in patients with uncontrolled asthma (UCAA and UNAA).

In healthy volunteers, the exit time distribution curve has a tendency to overlap with the shuffled curve. This indicates that rare fluctuations in respiratory rate tend to appear randomly in healthy subjects. In contrast, asthmatic patients show a significant deviation from their corresponding shuffled curves. This means that at any given exit time, it is less likely to observe a defined accelerating event compared to the shuffled data. More intuitively, this shows that it is more likely that a rare event (e.g. tachypnea) is followed by further, similar rare events in patients with asthma compared to healthy volunteers.

### Effect of asthma on *τ_m_* in breathing time-series

**Figure 3:**
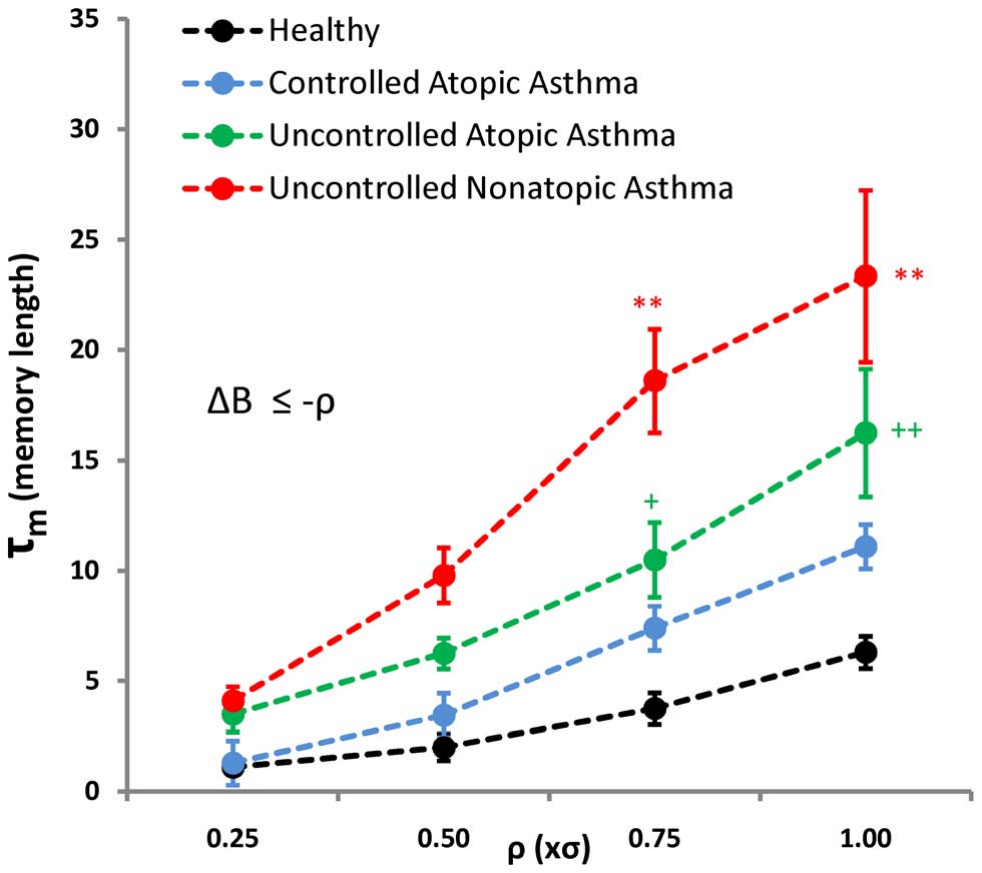
Comparison of the memory length (*τ_m_*) for observing accelerating events with varying thresholds (ρ = 0.25σ, 0.5σ, 0.75σ and σ, ΔB≤−ρ) between healthy volunteers and patients with different types of asthma. Respiratory inter-breath interval time-series were obtained from 10 healthy volunteers, 10 patients with controlled atopic asthma (CAA), 10 patients with uncontrolled atopic asthma (UCAA) and 10 patients with uncontrolled non-atopic asthma (UNAA). Data are presented as mean ± standard error of mean. *P<0.05, **P<0.01 in comparison with healthy subjects, CAA and UCAA. +P<0.05, ++P<0.01 in comparison with healthy volunteers (Bonferroni *post-hoc* test).

In figure 2, we can observe that in both patients and healthy volunteers, the difference between the two distributions occurs only in small *τ* regions, and after that, the two distributions merge. We call this interval *τ_m_*, which can be interpreted as memory length. As shown in this figure, the value of *τ_m_* is larger in asthmatic patients compared to healthy volunteers. Likewise, UNAA patients exhibit longer *τ_m_* than UCAA and CAA groups. In order to show this difference quantitatively, *τ_m_* values were computed for different ρ levels (ρ = 0.25σ, 0.5σ, 0.75σ and σ). As shown in figure 3, there is a significant difference in *τ_m_* between experimental groups (F_group_ = 35.0, P<0.001, two-way ANOVA). Moreover, patients with uncontrolled asthma (UCAA and UNAA) show significantly higher *τ_m_* than patients with controlled asthma or healthy volunteers. Statistical analysis also shows that *τ_m_* increases (regardless of the groups) when ρ is set for rarer events (F_ρ_ = 44.1, P<0.001, two-way ANOVA). The level of ‘rarity’ of the jump in a time-series clearly depends on the definition of *ρ* and figure 3 shows the memory length as the function of rarity of the jump. This finding indicates that the system has simple low order dynamics and short memory around its average (e.g. ρ = 0.25σ), but has more complex high order dynamical behavior (and longer memory length) around more infrequent fluctuations (e.g. ρ = σ).

### Inverse statistical approach in heartbeat time-series in patients with cirrhosis

**Figure 4:**
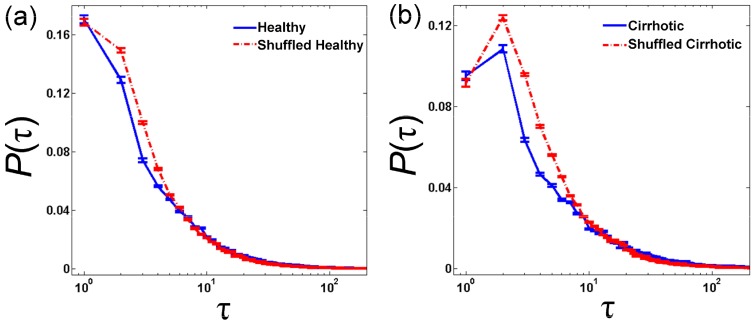
The probability distribution of the normalized exit times (τ) needed to observe a decelerating event that is ρ = σ second slower than a given point (t) within the time-series (ΔB = B_t+τ_−B_t_ ≥σ). Cardiac inter-beat interval time-series were obtained from (a) 41 healthy volunteers and (b) 93 patients with liver cirrhosis. Blue solid lines represent probability distribution of *τ* of original time-series and red dash-dot lines correspond to their shuffled time-series. Data are presented as mean ± standard error of mean.

In order to compare the inverse statistical properties and memory length of the cardiac inter-beat interval time-series in healthy subjects and patients with cirrhosis, 41 healthy volunteers and 93 patients were studied. Figures 4 shows the exit time distribution for the levels of ρ = σ in healthy volunteers and cirrhotic patients. These curves show the probability distribution of the time needed to observe heartbeat ρ seconds slower (*ΔB≥ρ*). As shown in this figure, the original heartbeat time-series is more similar to the shuffled time-series in healthy volunteers compared to cirrhotic patients. This suggests that at any given exit time, it is less likely to observe a defined decelerating event in comparison with the shuffled data set in patients with cirrhosis. In other words, it is more likely that a decelerating event (e.g. bradycardia) is followed by further, similar events in patients compared to healthy volunteers.

### Effect of cirrhosis on *τ_m_* in heartbeat time-series

**Figure 5:**
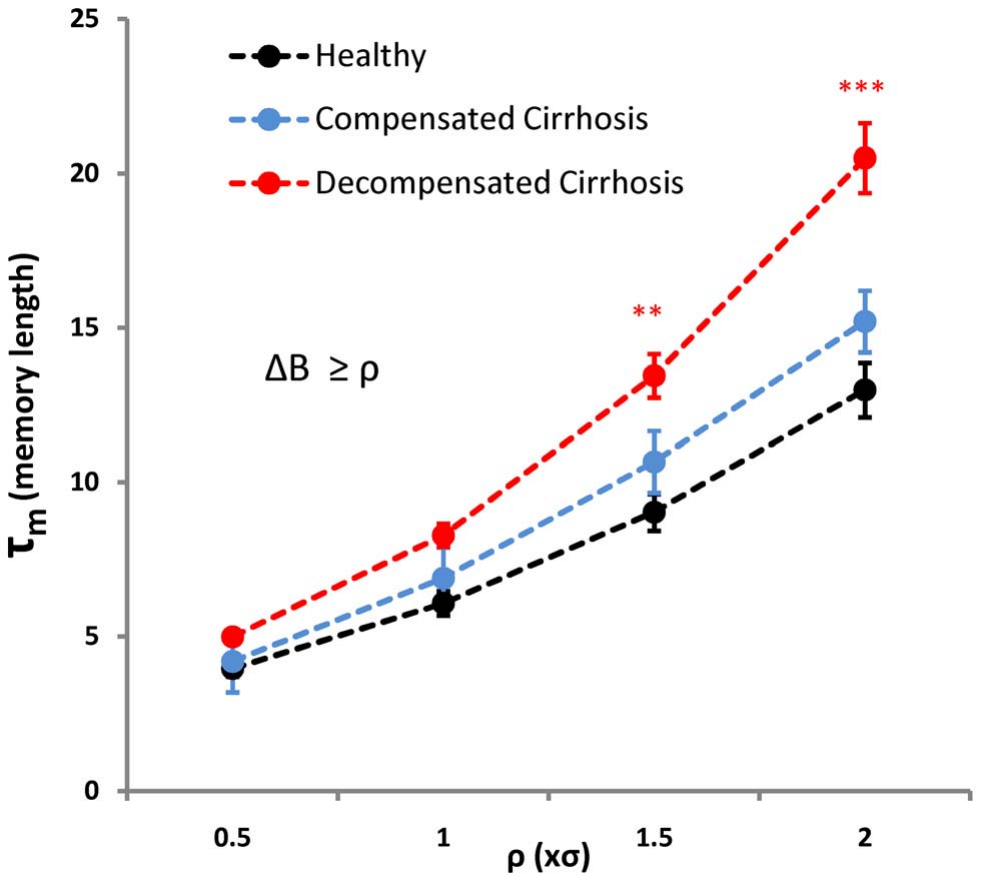
Comparison of the memory length (*τ_m_*) for observing decelerating events with varying thresholds (ρ = 0.5σ, σ, 1.5σ and 2σ, ΔB≥ρ) between healthy volunteers and patients with different degree of hepatic failure. Cardiac inter-beat interval time-series were obtained from 41 healthy volunteers, 26 patients with compensated cirrhosis and 67 patients with decompensated cirrhosis. Data are presented as mean ± standard error of mean. *** P<0.001 in comparison with healthy volunteers (at ρ = 1.5σ and 2σ) and patients with compensated cirrhosis (at ρ = 2σ).

We categorized cirrhotic patients into two groups based on the degree of hepatic impairment (26 patients with compensated cirrhosis and 67 with decompensated cirrhosis). We then calculated *τ_m_* values for different ρ levels (ρ = 0.5σ, σ, 1.5σ and 2σ). The average *τ_m_* is higher in cirrhotic patients compared to healthy volunteers (figure 5). In addition, cirrhotic patients with decompensated liver disease exhibited longer *τ_m_* length than patients with compensated disease. Two-way ANOVA showed that there was a significant difference in *τ_m_* between the study groups (F_group_ = 22.7, P<0.001) and *τ_m_* increased (regardless of the groups) when ρ was set higher (F_ρ_ = 120.7, P<0.001). Based on these results, it would appear that there is a longer memory length in the patients’ heartbeat time-series. This may suggest that sudden decelerating events could potentially affect the heart rhythm of cirrhotic patients for longer than healthy volunteers. Similarly to breathing dynamics, our heartbeat rhythm data indicate that the system has a short memory around its average region, but a longer memory length following more scarce fluctuation; a phenomenon which is more prominent in patients with decompensated cirrhosis.

## Discussion

The analysis of the behavior of a complex natural system is usually based on the assessment of the nonlinear interactions, as well as the determination of the characteristics of the fluctuating forces. This immediately leads to the problem of retrieving a stochastic dynamical system from the original data. A basic ingredient for such analysis can be the detection of a Markov property that can be observed in real systems above a certain length scale [4]. In the present study, we used an inverse statistical approach and computed the memory length (*τ_m_*), which gives an estimate for a transition from a Markov to a non-Markov process over relatively short physiological time-series (8 min ECG, 1 h respiratory cycle). As discussed above, this method might give information on memory and ‘controllability’ of physiological systems. Although the concept of memory and controllability has widely been used in engineering and physical sciences, it has not been widely investigated in autonomic physiological systems such as cardio-respiratory cycles. In the present study we provided examples of two medical conditions (asthma and cirrhosis) which are associated with alterations in the complexity of cardio-respiratory fluctuations [22], [24]. We observed that inverse time-series in healthy subjects are closer to shuffled time-series, and provided empiric evidence to show that there is a longer memory length in pathological conditions for both cardiac and respiratory time-series.

Asthma is still a major health problem for all age groups. For most patients, control of asthma can be achieved with the available drugs. However, in a small group of patients, asthma cannot be controlled and may cause death. Recent studies have shown that a systemic approach to respiratory dynamics may give novel insight into the pathophysiology of asthma attacks and respiratory failure [24]. Frey *et al*. analyzed the time-series of peak expiratory flows in patients with asthma and showed that the inter-breath interval time-series exhibits long-range correlations that change significantly with disease severity [24]. The physiological origins of these correlations are currently unknown, and the association between disease severity and respiratory cycle dynamics remains to be understood. The use of inverse statistical analysis has the advantage of translating the complexity of physiological rhythms into a probability of observing joint rare events. For instance, our results show that it is more likely that an accelerating rare event is followed by further, accelerating rare events in pathological situations such as uncontrolled asthma. The pathophysiological feature of life-threatening asthma is air trapping (dynamic hyperinflation), which impairs gas exchange and alveolar ventilation [16]. Air trapping mainly arises following episodes of continuous rapid respiratory rate during increased resistance to expiratory gas flow [25]. It is also known to clinicians that increased expiratory times are beneficial in the management of patients with severe asthma attacks [25]. Our data show that patients with uncontrolled asthma exhibit longer *τ_m_* compared to healthy subjects and patients with controlled atopic asthma. Translating this concept into clinical medicine, a sudden accelerating event could potentially affect the respiratory rhythm of asthmatic patients for longer than healthy controls. In other words, if a rare event (e.g. sudden acceleration of respiratory rate) occurs in healthy subjects, they tend to ‘forget it’ earlier than patients with asthma. Furthermore this memory is exaggerated in patients with uncontrolled asthma (UCAA and UNAA), a phenomenon which may make them more susceptible to develop dynamic hyperinflation. Apart from the importance of breathing dynamics in pathophysiology of asthma, respiratory rhythm fluctuation analysis has the potential to be used for noninvasive risk assessment for development of asthma episodes [26]. The concept of memory has a direct implication in quantification of controllability in physiological systems such as cardio-respiratory system [10]. These concepts, however, have not been used for risk assessment in patients with asthma. Future investigations may evaluate the diagnostic or prognostic value of these novel concepts in predication of acute exacerbation of asthma. In present study we focused on development of a method for quantification of memory in physiological time-series in health and disease. Our report does not explore the mechanism of increased memory length of respiratory time-series during asthma. Future studies might look to understand the mechanism and implications of present findings.

We also looked at cardiac inter-beat interval time-series in patients with cirrhosis. Cirrhosis is associated with a functional cardiomyopathy which is defined as cardiac chronotropic/inotropic incompetence under conditions of physical or pharmacological stress [21]. Previous reports have shown that β-adrenergic-dependent cardiac accelerating mechanisms are impaired in this patients’ population [21]. The exact prevalence of cirrhotic cardiomyopathy remains unknown but it is believed that the majority of patients with cirrhosis who have reached Child-Pugh stages B or C (or decompensated cirrhosis) exhibit clinical features of cirrhotic cardiomyopathy [21]. In the present study we showed that there is a significant elevation of *τ_m_* for decelerating cardiac events in patients with decompensated liver cirrhosis compared to healthy people and patients with compensated cirrhosis. This may indicate that a sudden decelerating event could potentially affect the cardiac rhythm of patients with advanced cirrhosis for a longer duration than healthy subjects or patients with compensated cirrhosis. Thus, if a decelerating cardiac event occurs in a healthy person, it will be ‘forgotten’ earlier than in a patient with advanced cirrhosis. Whether or not this phenomenon is part of cirrhotic cardiomyopathy, continuous bradycardia represents a clear disadvantage for a cirrhotic patient in unstable circulatory conditions. We also looked at the memory length to observe accelerating rare events in the heartbeat time-series of healthy subjects and patients with cirrhosis. However we were unable to find a significant difference between the two groups when *ΔB≤−ρ* (data not shown). Although this finding goes along with impaired cardiac responsiveness to accelerating adrenergic stimulation in cirrhosis [21], [22], it also indicates that prolongation of memory in cardiac rhythm in cirrhosis is not symmetric in terms of observing both accelerating and decelerating rare events.

Liver cirrhosis is a complex disease and is associated with peripheral vasodilatation, volume overload as well as cardiomyopathy [21], [27]. Peripheral vasodilatation reduces cardiac afterload that masks clinical sign and symptoms of cardiomyopathy in patients with cirrhosis. Therefore, cirrhotic cardiomyopathy remained an academic curiosity until recently, initially because it appeared to have little clinical relevance [27]. However, the widespread use of orthotopic liver transplantation and its associated stresses on the cardiovascular system have highlighted this type of cardiomyopathy [27], [28]. Heart failure has emerged as an important cause of morbidity and mortality in the liver transplant recipient [27], [28]. Due to lack of sensitive and noninvasive diagnostic tests, recognition of cirrhotic cardiomyopathy is difficult prior to liver transplantation. Echocardiography has been used for noninvasive diagnosis of cardiac dysfunction. However, due to volume overload, most of the echocardiographic cardiac indices cannot be used for the evaluation of systolic function in cirrhotic patients. The diagnosis of cirrhotic cardiomyopathy is still a challenge for clinicians, as it is mainly based on exercise or pharmacological stress tests, which are not always feasible. There is a hope that complexity science can pave the way for development of novel noninvasive techniques for diagnosis of cirrhotic cardiomyopathy based on mathematical analysis of physiologic signals. To the best of our knowledge, our method of observing memory in physiological time-series is novel and may be applied to translational research as well as clinical medicine (i.e. the diagnosis of cirrhotic cardiomyopathy). Future studies may provide useful insight into the application of the concept of “memory in complex physiological rhythms” in clinical practice.

As described above *τ_m_* indicates the minimum time interval over which the data can be considered as a Markov process in the prediction of observing a rare event. Thus *τ_m_* is connected to the limitations we face in predicting the future of a time-series. Increased *τ_m_* of cardio-respiratory time-series system in pathological conditions shows that the future of the time-series might be more predictable in disease. Although this is a disadvantage for an adaptive system in response to an ever-changing environment, it explains why it is more likely to observe predictable patterns in cardio-respiratory rhythm during pathologic conditions (e.g. Cheyne-Stokes breathing in patients with congestive heart failure) [29].

Memory is a concept that has been used in a variety of disciplines. In the present study, the concept was ‘quantified’ by determining the time scale over which rare fluctuations do not appear randomly within a physiological time-series. We also provided empiric data to show that rare fluctuations in both cardiac and respiratory cycles are ‘forgotten’ quickly in healthy subjects while their memory is kept for longer in pathological conditions. This report corroborates the concept of “dynamical diseases” [30] and the common experience that sometimes ‘it is best to forget’!

## Supporting Information

File S1 Contains: Appendix I: Glossary of the technical terms used in present study. Appendix II: Mathematical basis for the extraction of ‘memory length’ from inverse statistical analysis of a given time-series.
(DOCX)
