# Supplementary material for: Quantifying Memory in Complex Physiological Time-Series
Source: PLoS One. 2013 Sep 5;8(9):e72854. doi: 10.1371/journal.pone.0072854 (PMC3764113; doi:10.1371/journal.pone.0072854)
Supplement: File S1 — Contains: Appendix I: Glossary of the technical terms used in present study. Appendix II: Mathematical basis for the extraction of ‘memory length’ from inverse statistical analysis of a given time-series. (DOCX) [file pone.0072854.s001.docx]

**Appendix I**

**Glossary:**

Exit time: The waiting times (τ) needed to achieve a predefined difference in a time-series.

N-step joint probability: The probability to observe a defined event n steps following another defined event.

Markov length: Markov length is the minimum time interval (length scale) over which the data can be considered as a Markov process.

Markov process: A random process whose future probabilities are determined by its most recent values. A Markov process can be thought of as a “memoryless” stochastic process.

Stationary process: A process where its statistical properties do not vary with time is called a stationary process. Processes whose statistical properties do change are referred to as non-stationary.

**Appendix II**

This appendix is dedicated to reveal that memory length is the block size of dynamics, and all larger block sizes can be reconstructed by some multiplication of shorter ones.

Suppose a non-stationary time-series *B_t_*, the probability of the first passage time of the level *ρ* (i.e. observing jump *ΔB= ρ* at the *τ* interval after the time *t*) is:

1. $p(B_{\tau+t}-B_{t}>\rho, B_{\tau+t-1}-B_{t}<\rho, \ldots, B_{t+1}-B_{t}<\rho)$

It means that for an arbitrary time *t*, the corrected time-series subtracted from *B_t_* level, should be below the level *ρ* in *t +τ-1* steps, and then jumps up the level *ρ* in step *t +τ*. In other word, we want to extract the probability of waiting time which takes to observe a jump *ΔB = ρ.* Hence, there should be a jump at step *τ*, and all steps before that should have a smaller jumps.

Now, we make the shuffled time-series *B_t_^*^*. Again, the first passage time probability is defined as:

1. $p\left( B_{\tau+t}^{*}-B_{t}^{*}>\rho, B_{\tau+t-1}^{*}-B_{t}^{*}<\rho, \ldots, B_{t+1}^{*}-B_{t}^{*}<\rho\right)$

But because of the shuffling nature, this joint probability can be separated in this manner:

1. $p\left( B_{\tau+t}^{*}-B_{t}^{*}>\rho) p(B_{\tau+t-1}^{*}-B_{t}^{*}<\rho) \ldots p(B_{t+1}^{*}-B_{t}^{*}<\rho\right)$

According to our observations, this distribution collapses (in the range of error bars) on the original one after a time step *τ_m_*. Hence, Formula 1 and 3 are equal for *τ > τ_m_*. Therefore, holding this condition, someone can calculate the joint probability Formula 1 from separated probabilities. In other word, the largest joint probability of first passage times in the original time-series which are not reducible to the simple part, is in the size of *τ_m_*. And the largest joint probability size which is necessary to explain the system jumps *ΔB = ρ* is *τ_m_*.
